# Exploring the Sensory Typicity of Timorasso Wines: Physicochemical and Sensory Characteristics of Seven Consecutive Vintages

**DOI:** 10.3390/foods14040591

**Published:** 2025-02-11

**Authors:** Maria Alessandra Paissoni, Micaela Boido, Pietro Margotti, Simone Giacosa, Susana Río Segade, Vincenzo Gerbi, Luca Rolle, Christoph Schuessler, Rainer Jung, Doris Rauhut, Andrii Tarasov

**Affiliations:** 1 Department of Agricultural, Forest, and Food Sciences, University of Torino, Corso Enotria 2/C, 12051 Alba, Italy; micaela.boido@unito.it (M.B.); simone.giacosa@unito.it (S.G.); susana.riosegade@unito.it (S.R.S.); vincenzo.gerbi@unito.it (V.G.); luca.rolle@unito.it (L.R.); 2 Interdepartmental Centre for Grapevines and Wine Sciences, University of Torino, Corso Enotria 2/C, 12051 Alba, Italy; 3 Department of Enology, Hochschule Geisenheim University (HGU), Von-Lade-Straße 1, 65366 Geisenheim, Germany; pmargotti@gmail.com (P.M.); Christoph.Schuessler@hs-gm.de (C.S.); Rainer.Jung@hs-gm.de (R.J.); 4 Department of Microbiology and Biochemistry, Hochschule Geisenheim University, Von-Lade-Straße 1, 65366 Geisenheim, Germany; Doris.Rauhut@hs-gm.de

**Keywords:** Timorasso, white grape variety, sensory analysis, kerosene aroma, empyreumatic, minerality, *Vitis vinifera* L. grapes

## Abstract

‘Timorasso’ is an autochthonous, non-aromatic white grape variety cultivated mainly in the southwest of the Piedmont region (northwestern Italy). The sensory profile of wines produced from this variety evolves greatly with aging. In this study, 31 wines from 2015–2021 vintages were analyzed to investigate changes in sensory descriptors at various stages of aging and their correlation with physicochemical properties (wine basic parameters, color, and total polyphenols) and sensory-perceptual typicity. A sensory analysis was conducted by a panel of experts, who were asked to indicate the in-mouth and aroma descriptors. The aroma-related terms were analyzed as individual descriptors or grouped in “Categories”. Moreover, the panel rated the *Color*, *In-Mouth*, and *Aroma typicity* of these wines. ‘Timorasso’ based wines were found to have, on average, a relevant alcohol content (14.20 ± 0.56% *v*/*v*), moderate acidity (5.8 ± 0.6 g/L), and low pH (3.19 ± 0.09). In fact, Timorasso wines were sensorially identified in terms of citation frequency with the in-mouth descriptors *acidity* (32.9%), *sapidity* (25.5%), and *minerality* (17.4%). The aroma of younger wines (2 years of aging) was characterized by “Green”, “White flowers”, “White pulp fruit”, and “Citrus”. In general, the most cited aroma category was “Kerosene” (27.9%), distinguishing wines with 5–6 years of aging. “Kerosene” category correlated with *Aroma typicity* (*p* < 0.001), as well as with “Balsamic” (10.8%, *p* < 0.01) and “Empyreumatic” (5.5%, *p* < 0.05) aroma categories.

## 1. Introduction

The varietal identity of many wines has been the subject of research in recent years, and the main Italian cultivated varieties have been widely investigated [1,2,3,4,5]. Despite this, Italy’s rich ampelographic heritage of about 640 winegrape varieties and more than 400 PDO (Protected Designations of Origin) wines with a production volume of 18.7 million hectoliters in 2023 [6] remain only partially explored. Thus, many high-value local varieties and associated PDO wines require more detailed sensory and physicochemical insights.

The Italian grape (*Vitis vinifera* L.) cultivar ‘Timorasso’ is a white autochthonous, non-aromatic variety primarily grown in the Piedmont region (northwest Italy), specifically in the southwestern rural area of the village of Tortona (Alessandria province). In the 19th century, ‘Timorasso’ was a very important variety in this area, accounting for a quarter of the surface cultivated with white grapes [7]. Later, the ‘Timorasso’ cultivation was almost completely abandoned due to phylloxera and the post-war depopulation of rural areas as people moved to industrial cities [8]. ‘Timorasso’ yield is inconsistent due to its tendency to flower abortion and millerandage, and, in general, it is lower in comparison to other varieties commonly cultivated in the area, such as the widespread ‘Cortese’ [9,10]. By the mid-1980s, “Timorasso’ was almost extinct, but thanks to the initiative of several local winemakers, ‘Timorasso’ was brought back to the forefront of the Colli Tortonesi PDO (Product Designation of Origin) appellation [11]. Since its rediscovery, there has been considerable growth in the area planted with this cultivar, rapidly increasing to a total of 330 hectares by 2023 [12].

The Colli Tortonesi PDO is a protected area historically dedicated to the ‘Timorasso’ grapes. The regulations provide for five main categories of Timorasso wines: “Colli Tortonesi Bianco” or “Frizzante”; “Colli Tortonesi Timorasso”; “Colli Tortonesi Timorasso Riserva”; “Terre di Libarna Bianco” or “Spumante”; and “Terre di Libarna Timorasso”. Libarna is a sub-zone in the larger area of Colli Tortonesi. When Timorasso is indicated on the label, at least 95% of Timorasso grapes must be used for wine production. Usually, the Timorasso wine release date is one year after the harvest (1 of November for “Colli Tortonesi” Timorasso and 1 of September for “Terre di Libarna” sub-zone), whereas the “Riserva” label is designated for wines that have undergone a minimum of 21 months of aging, starting from 1 November following the harvest [13]. Recently, the Consortium of Colli Tortonesi PDO decided to invest in the appellation *Derthona*, the Latin name of the Tortona town, linking ‘Timorasso’ to its territory and specifying it as a new sub-zone within the Colli Tortonesi PDO. Besides Colli Tortonesi PDO, it is allowed to produce Timorasso wines in the Lombardy region in several PGIs (Protected Geographical Indications). Other countries have also shown interest in this cultivar, such as France (Champagne region), the USA (California), Chile, and Ukraine [10].

In the past, the ‘Timorasso’ variety was not very popular, producing basic quality wines that were easy to drink and had a short shelf life [9]. Nowadays, after its rediscovery and the implementation of knowledge about the varietal potential and the application of new winemaking technologies, Timorasso is considered a wine with great aging potential [10,14]. This is supported by its significant alcohol content and low pH, although relevant data are lacking in the current scientific literature. Considering the aroma composition, ‘Timorasso’ is a non-aromatic white grape, which means that varietal aromas are not predominant in young wines. On the other hand, other categories of aromas are well perceivable in Timorasso wines, such as secondary aromas coming directly from fermentation and tertiary aromas resulting from wine aging and maturation. Recently, Laureati and colleagues [15] conducted a preliminary investigation on the sensory identity of monovarietal Timorasso wines from the 2018 vintage. The wines were tasted after four years of storage, revealing multifaceted production and styles even within the same vintage, ranging from fruity notes to solvent/fuel descriptors. Three very heterogenous macro-clusters were identified: one with lemon and peach aromas, low acidity, body, and low color; another with ethereal aromas and high color hue; and the last one had vegetal notes and high acidity [15]. Thus, different production strategies resulted in various Timorasso wine styles. A common descriptor was saltiness, which characterized all Timorasso wines studied [15]. However, the physicochemical characterization of Timorasso wines, along with the investigation of their aging potential from sensory and analytical perspectives, are still unexplored.

The aromatic descriptors of Timorasso wines, found in the professional (non-scientific) literature, are numerous. They do not always coincide and can be separated for young and aged wines. Young Timorasso wines have been described with herbaceous, almond paste, and vegetal notes [9] or pear, citrus, and melon, as well as acacia flowers and tomato leaves [16]. Aroma descriptors for aged/developed Timorasso wines included flintstone and toasted nuts [9], “hydrocarbons”, petroleum, ripe pear, and honey [9,16].

This study represents the first stage of our investigation of Timorasso wines. Its aim was to perform a screening of commercially produced wines made from ‘Timorasso’ grapes of different vintages to investigate changes in sensory descriptors at various stages of wine aging, obtaining valuable data for more comprehensive future research. In addition, the physicochemical analyses of the wines were performed to understand their main physicochemical parameters, color, and phenolic characteristics. Correlations between the instrumental parameters and sensory descriptors were explored. Finally, the expert panel was asked to rate the typicity of Timorasso wines (considering the wine age), and these results were correlated with individual descriptors. Experts inside a specific *terroir* are familiar with a certain product and accustomed to its habitual consumption and are recognized as a useful tool for the evaluation of the sensory perceptual typicity of specific PDO wines [17,18,19], allowing its characterization.

## 2. Materials and Methods

### 2.1. Wine Selection

The wines used in this study were provided by the producers of the Consorzio dei Colli Tortonesi DOC (Denominazione di Origine Controllata, Italian PDO, Tortona, Italy). They were asked to propose one or two samples of varietal Timorasso wines from their production that they considered representative of the global conceptual typicity [17]. A vintage range was set from 2015 to 2021, and 31 wines from 21 producers were collected (Table S1): 2021 (4 wines), 2020 (6 wines), 2019 (5 wines), 2018 (6 wines), 2017 (6 wines), and 2016 and 2015 (2wines each). The producers represented twelve districts in the Alessandria province and one in the Cuneo province (producing Timorasso wine using grapes from the Colli Tortonesi area). Twenty-four wines were labeled “Colli Tortonesi Timorasso DOC”, two were “Colli Tortonesi Timorasso Riserva DOC”, one was “Colli Tortonesi Terre di Libarna DOC”, and four were “Vino bianco” wines without PDO but made from at least 95% ‘Timorasso’ grapes. Of the 31 wine samples analyzed, 11 were sealed with agglomerated cork closures, 10 with synthetic stoppers, 7 with single-piece natural corks, and 3 with screw caps. In this way, an attempt has been made to consider the Timorasso wines’ heterogeneity.

### 2.2. Physicochemical Analysis

A 150 mL sample was taken from each bottle and used for the analysis of basic wine parameters, color, and polyphenolic compounds. Values are expressed as averages of two measurements. pH was analyzed using a calibrated pH meter InoLab 730 (WTW, Weilheim, Germany) (OIV-MA-AS313-15), and total acidity was analyzed by titration with sodium hydroxide solution (0.1 mol/L) and bromothymol blue as an indicator (OIV-MA-AS313-01), as reported by the International Organization of Vine and Wine (OIV) [20]. Organic acids, sugars, glycerol, and ethanol were determined by HPLC-UV-RI, as described by Giordano et al. [21]. A 1260 Agilent HPLC system was equipped with a UV set at 210 nm and a refractive index detector (Agilent Technologies, Santa Clara, CA, USA). Chromatographic separation was performed using a 300 mm × 7.8 mm i.d. Aminex HPX-87H cation exchange column and a cation H+ Microguard cartridge (Bio-Rad Laboratories, Hercules, CA, USA) at 65 °C. The mobile phase was an isocratic flow of 0.0065 mol/L H_2_SO_4_ at 0.8 mL/min. Samples were injected after being filtered through 0.45 μm PTFE membrane filters.

The wine dry extract was analyzed according to the method OIV-MA-AS2-03B by wine distillation using the DEE Gibertini distiller (Novate Milanese, Italy) and specific gravity measurements of wines and distillates by the DMA1001 Anton Paar densimeter (Graz, Austria). To calculate the net dry extract, the residual sugars resulting from the HPLC analysis (glucose and fructose) were subtracted from the total dry extract calculated by interpolating the specific gravity data to values from standardized tables [20].

A UV-1800 spectrophotometer (Shimadzu Corporation, Kyoto, Japan) was used for the chromatic characteristics and total polyphenols (TPI) analysis. To determine TPI, wines were diluted 10 times with distilled water, and then the absorbance was measured from 230 to 400 nm in a 10 mm quartz cuvette. The absorbance at 280 nm was recorded, and the results were expressed as mg/L (-)-epicatechin using an external calibration curve. To determine chromatic characteristics according to CIELab (method OIV-MA-AS2-11), spectra from 380 to 780 nm were recorded in a 10 mm plastic cuvette. In specific, L* is clarity (L* = 116(Y/Y_n_)^1/3^ − 16), a* is a red/green color axis (a* = 500[(X/X_n_) − (Y/Y_n_)]), b* is a yellow/blue color axis (b* = 200 − [(Y/Y_n_)^1/3^ − (Z/Z_n_)^1/3^], and their derived magnitudes are chroma (C*= √(a*^2^ + b*^2^)) and tone (H* = tg^−1^ (b*/a*)). The L*, a*, and b* coordinates were also converted to 24-bit RGB color space for visualization purposes [3]. Absorbance at 420 nm (A420) was considered an indicator of the yellow component of the wine color.

### 2.3. Sensory Analysis

The sensory analysis of the 31 Timorasso wines was carried out by a panel of 20 experienced judges [22]: established winemakers, enology graduates, and wine-science researchers and professors involved in winemaking and/or wine sensory evaluation. They were recruited among Timorasso wine producers in the area of the Colli Tortonesi PDO and among employees of the University of Turin. All judges were asked to have previous experience in sensory analysis of both a tasting procedure similar to the one described below and wines made from ‘Timorasso’ grapes. The tasters were between 23 and 65 years old, and 25% of them were women. The judges signed an informed consent to participate in the tasting after explaining the wine samples’ nature, the tasting procedure, the aim of the research, and the data treatment. The tasting took place on 19 May 2023 in the sensory room of Vigneti Repetto winery (Sarezzano, Italy).

Each bottle/sample was anonymized (using a black cover) and coded with a three-digit number. The tasting was divided into 5 sessions with a 20 min break between them: six wines per session, except for the 5th session with 7 wines. The wines were served at 16–18 °C and randomized for each judge within the sessions. The judges were informed about the vintage range for each session: the first session included wines from the recent vintages of 2021 and 2020; each subsequent session comprised increasingly older wines, with the final session having 2017 to 2015 vintages.

Paper questionnaires were given to the judges (Figure S1), and they were asked to evaluate, firstly, the wine’s *Visual color hue*, *Color typicity*, *Aroma typicity*, and *In-Mouth typicity* using 0–10 continuous scales. The *Liking* parameter was assessed on a 100-point scheme, defined as follows: 95–100 as great/exceptional wine; 90–94 as excellent; 85–89 as very good; 80–84 as good; 75–79 as adequate; and <75 as a low score requiring justification indicating a defect/fault. This rating was in accordance with the Wine Spectators 100-point scale (https://www.winespectator.com/articles/scoring-scale, accessed on 14 May 2023).

Finally, the judges were asked to list descriptors for each wine in terms of aromas (orthonasal and retronasal modalities together), taste, and mouthfeel (in-mouth sensations). Appropriate terminology for descriptors was proposed (Table S2), which aimed to reduce variability in answers [23]. The offered descriptors were selected according to the “Aroma Wheel” proposed by Noble et al. [24] and slightly modified according to descriptions of Timorasso wines found in technical and scientific sources [14,16]. The descriptors were pre-divided into three hierarchical levels: GROUP/“Categories”/*individual descriptors* (Table S2). The aroma-related GROUP and “Categories” were as follows: FRUITY (“White pulp fruits”, “Yellow pulp fruits”, “Tropical fruits”, “Citrus”), FLORAL (“White flowers”, “Other flowers”), VEGETAL (“Fresh (green)”, “Hay/herbs”, “Balsamic”), AGING/EVOLUTION (“Honey”, “Dried fruits”, “Nuts”, “Spices”, Toasted”), MICROBIOLOGICAL (“Yeast”), CHEMICAL (“Kerosene”, “Reduced”, “Empyreumatic”, “Chemical”), and OXIDIZED (“Pungent oxidation”, “Light oxidation”). Concerning the taste-related “In-Mouth”, it was divided into the “Categories” “Taste” (*acidity*, *bitterness*, *sweetness*) and “Others” (*astringency*, *body, texture*, *warmth alcohol*, *smoothness*, *sapidity/savoriness/saltiness*, *minerality*). The judges were asked to report one or more descriptors for each wine, and these had to be perceived evidently and strongly characterizing the analyzed samples. The list (Table S2) was read collectively before the tasting to ensure everyone comprehended the terminology used.

For sensory analysis, the 0–10 continuous scales were measured for the corresponding parameters. The individual descriptors indicated by the judges were marked as present/absent in Excel (Version 2406, Microsoft Corporation, Redmond, WA, USA). Then, citation frequencies (CF, in %) were calculated for each individual descriptor (1) and corresponding “Categories” (2) according to the following equations:

(1) $$Individual\;descriptor\;CF=\frac{number\;of\;individual\;descriptor\;occurrence}{number\;of\;judges\;\times \;number\;of\;wine\;samples}\times 100\%$$

(2) $$“Category”\;CF=\frac{number\;of\;“Category”\;occurrence}{number\;of\;judges\;\times \;number\;of\;wine\;samples}\times 100\%$$

For “Category”, the frequency may vary with respect to the sum of individual descriptors since, when both descriptors and categories were reported from the judges, they were counted as one occurrence.

### 2.4. Statistical Analysis

Statistical analyses were performed using RStudio statistic software 2023.12.1 (R Foundation for Statistical Computing, Vienna, Austria). For instrumental physicochemical analysis, the data were analyzed using Kruskal–Wallis for both the factor “sample” and the factor “vintage”. If there were significant differences (*p* < 0.05), a Dunn test was performed to determine differences among vintages. Pearson correlation coefficients (r) were calculated, and *t*-tests were performed to establish significant correlations between *Color typicity* and CIELab parameters and between *In-Mouth typicity* and basic wine parameters and total polyphenols.

The scale data were analyzed using two-way ANOVA with the sample as a fixed factor and judges as random. Data are expressed as mean ± standard deviation of the judges’ sensory evaluations (n = 20 for *Visual color hue* and *Liking*, n = 18 for the typicity evaluations). The different number of judges for *Liking* and typicity is explained by the fact that two professionals did not consider themselves experts in Timorasso-based wine production, and therefore, they were not included in the typicity evaluation. The results for aroma and taste (“In-Mouth”) descriptors were obtained from the entire panel (n = 20). Pearson correlation (*r*) was used for evaluating the correlation of typicity *with individual descriptors*/“Categories”. Statistically significant descriptors were assessed with Cochran’s Q test [25]. Correspondence analysis (CA) was performed using the R package Factominer [26] for both *individual descriptors* with a cut-off threshold of 1% and for “Categories” of descriptors.

## 3. Results and Discussion

### 3.1. Physicochemical Parameters of the Wines

**Figure 1:**
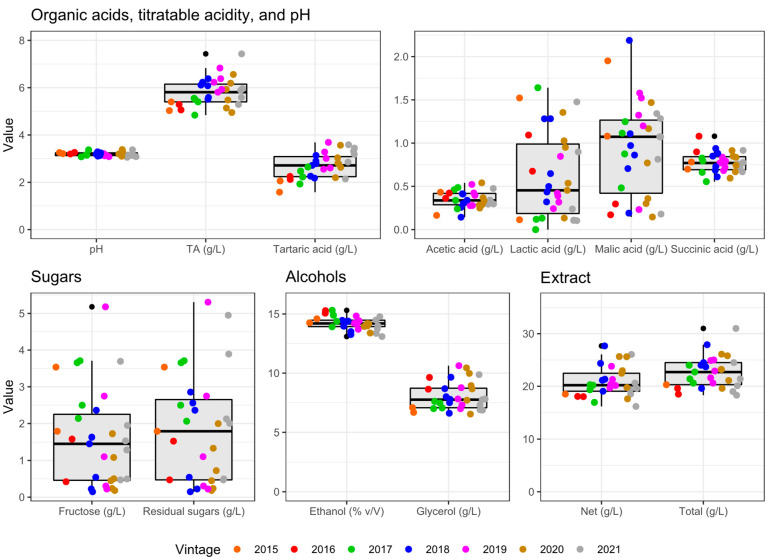
Basic physicochemical parameters of the 31 Timorasso wines used in the study. TA = total acidity, pressed as g/L of tartaric acid.

Timorasso wines were characterized by a relatively low pH (range 3.06–3.38 and average 3.19) and moderate total acidity (range 4.8–7.4 g/L and average 5.8 g/L—expressed in tartaric acid) (Figure 1 and Table S3). These values are in line with those of white Italian monovarietal wines [27], which have an average total acidity of 5.7 g/L. The average ethanol level of 14.20% (*v*/*v*) in Timorasso wines was higher than the alcohol content in Italian monovarietal wines on average (13.13%, *v*/*v*) [27]. The found values confirm the ability of ‘Timorasso’ grapes to accumulate sugars well while maintaining a good content of organic acids. This agrees with the data reported by Raimondi et al. [9], who compared ‘Timorasso’ grapes with other typical white varieties of south Piedmont such as ‘Cortese’ and ‘Favorita’. Many (about 70%) of the studied wines did not perform or only partially underwent malolactic fermentation, showing an average content of malic acid and lactic acid of 0.94 g/L and 0.62 g/L, respectively, thus increasing the overall perception of acidity. In this case, no differences were found depending on the vintages due to a higher impact of the winemaking strategy adopted by each winery. The tartaric acid content in older vintages was lower (*p* < 0.05), which may be due to initial harvest parameters and/or its loss during the aging process, also reducing total acidity (*p* < 0.05). Glucose concentrations in all wines were under 2 g/L, and fructose content was usually below 3 g/L, with the exception of five wines with a maximum concentration of 5.18 g/L. Older vintages (2015–2017) had lower total acidity and tendentially higher ethanol content (e.g., up to 15.17% (*v*/*v*) in 2016), which could be explained by late harvest or seasonal conditions that may have affected the grape ripeness. Glycerol contents varied significantly among Timorasso samples, probably due to the different yeast strains used for fermentation, with an average of 8.06 g/L and a maximum of 10.63 g/L.

The wine color was analyzed sensorially (*Visual color hue* in the range from light yellow to orange, *Color typicity*, Table S4) and instrumentally (CIELab parameters, Table 1). According to the OIV-MA-AS2-11 method [20], the wine color can be described by reporting three attributes that characterize visual sensations, i.e., tonality, luminosity, and chromatism. Clarity is related to the luminosity and is represented by the L* parameter. Tonality corresponds to the real color, which is represented by the components a* (red–green) and b* (yellow–blue), while the intensity or level of color can be described using chromatism-derived values of H* (tone) and C* (Chroma). Tendentially, older wines were more intense in color, and 2016 vintage wines had a more orange hue compared to 2021 ones (both sensorially, Table S4, and instrumentally with higher A_420_ values, Table 1). At the same time, winemaking techniques such as prolonged skin maceration might have an impact on certain young wines. For example, 2017 vintage wines were among those with the lowest visual color hue, while one 2020 vintage wine made with skin maceration showed the highest hue value of all samples (Table S4). The calculated CIELab parameters b* and C* agreed with the sensory results. In general, the sensory and instrumental (CIELab and A_420_) results correlated well with each other, showing good reliability of the panel’s visual evaluation (Figure S1). The recent vintage wines (2020–2021) were significantly different from older vintage samples in terms of *Color typicity*. The judges preferred wines with a lighter hue, which is confirmed by the negative correlation between *Liking* and *Visual color hue* (*r* = −0.488, *p* = 0.005, Table S5). At the same time, *Visual color hue* and *Color typicity* were not correlated (*p* > 0.05, Table S5, Figure S2), which means that ‘Timorasso’-based wines can be perceived as “typical” with different color hues.

**Figure 2:**
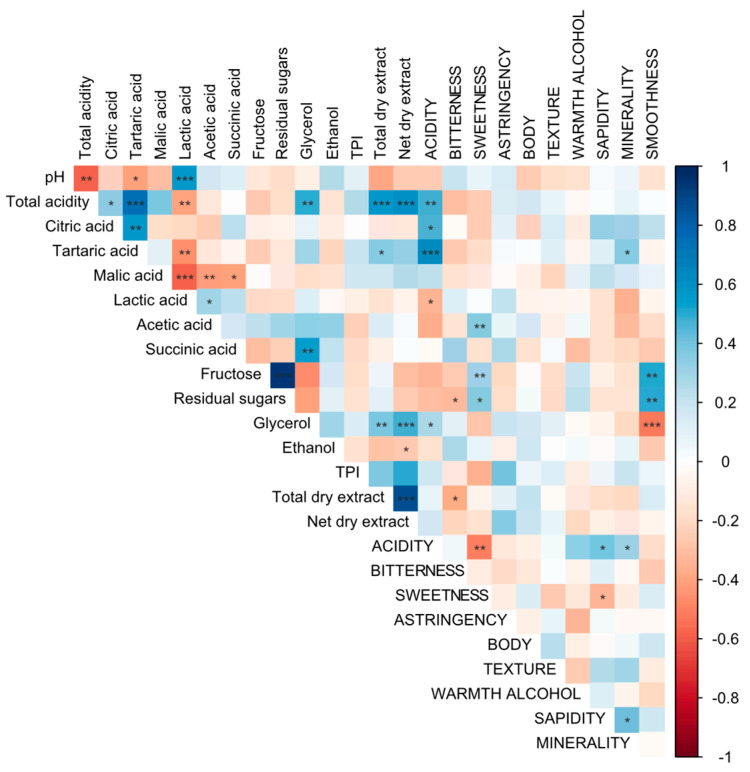
Pearson correlation plot of “In-Mouth” descriptors (in uppercase except TPI) and physicochemical parameters (where TPI = total polyphenols). Sign.: *, **, and ***, indicate significant correlation at *p* < 0.05, 0.01, 0.001.

Total polyphenols (Table 1) discriminated wines, but not consistently among vintages, with the highest values being found in 2015 and followed by 2020. The main differences may be related to production processes: skin contact in Timorasso wine production has been previously reported [15], although different maceration lengths, temperatures, and enzyme applications may be used, leading to different polyphenolic contents [28,29,30]. These compounds, along with others such as organic acids and glycerol, may contribute to the net dry extract that ranged from 17.6 to 27.7 g/L (Figure 1), reflecting the variability of production processes. These parameters (total polyphenols, net dry extract) are usually associated with astringency, bitterness, and wine body, but no significant correlations were found neither for total polyphenols nor for net dry extract with these descriptors (Figure 2). However, the *In-Mouth typicity* was strongly correlated with the overall *Liking* (*r* = 0.848, *p* < 0.001, Table S5), and it may rely on *individual descriptors*.

### 3.2. In-Mouth Descriptors and Their Correlations with Physicochemical Parameters and Typicity

**Figure 3:**
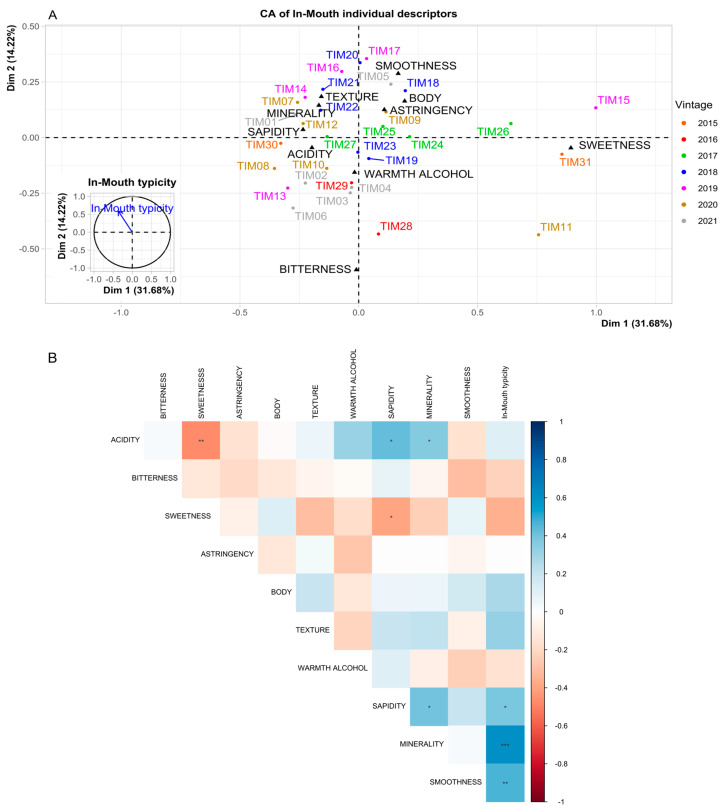
Correspondence analysis of the “In-Mouth” descriptors (**A**) and correlation plot of descriptors and *In-Mouth typicity* (**B**). Sign.: *, **, and ***, indicate significant correlation at *p* < 0.05, 0.01, 0.001.

Table 2 reports the “In-Mouth” descriptors resulting from the sensory analysis. *Acidity* was the most frequently mentioned descriptor (CF: 32.9%), while *astringency* and *texture* (7.1% and 8.4%, respectively) were less important when describing Timorasso wines, as usually expected for white wines in general. This is also consistent with the lack of correlation between physicochemical parameters and sensorially evaluated *astringency* (Figure 2). *Sapidity* (*saltiness/savoriness*) and *minerality* were also often reported by judges (25.5% and 17.4%, respectively). These descriptors strongly correlated with each other (Figure 3B), and both significantly correlated with *In-Mouth typicity* (*r* = 0.3892, *p* < 0.05 and *r* = 0.604, *p* < 0.001, respectively, Table 2). In addition, statistical analysis demonstrated that the studied Timorasso samples differed significantly in *sapidity* (*p* < 0.001).

The origin of *sapidity* and *minerality* descriptors requires further investigation. Previous work reported that saltiness (which is linked to the *sapidity* descriptor in our study) strongly characterized Timorasso wines [15]. *Minerality* assessment relies on different modalities, including aroma (orthonasal and retronasal) and taste (palate) [31,32]. In this study, we explored separately the concept of aroma perception of minerality due to the presence of certain volatile organic compounds (VOCs) and the concept related to in-mouth sensations. Concerning the latter, *sapidity* is considered a sensory dimension of *minerality* and may rely on the presence of cations and salts, particularly sodium chloride, as demonstrated in Sauvignon blanc wines from New Zealand [33]. However, the extent of this phenomenon is still an open question [32]. Sensory thresholds for cations and salts are rarely reached in wines [32], and this occurs mainly in regions close to the sea, which is not the case in the Colli Tortonesi area [15]. Also, the occurrence of these compounds was not aimed to be investigated in this study.

Another possible driver of the minerality perception in taste is higher acidity and the presence of increased amounts of certain organic acids in wine [32]. In the current study, *acidity* was also positively correlated with *minerality* and *sapidity* (Figure 3B), which, in turn, were related to high contents of organic acids and low pH. A correlation between *minerality* and the presence of specific organic acids (succinic, malic, tartaric acids) in relevant concentration has been found previously in Sauvignon blanc, Chardonnay, and Riesling wines from certain regions [31,32,33,34]. On the contrary, the occurrence of malolactic fermentation and, hence, lactic acid concentration was negatively correlated with minerality in previous studies [32,34]. In our case, a negative correlation was found, although not significant (*r* = −0.345, *p* > 0.05), probably due to heterogeneity of the results in terms of malolactic fermentation.

A negative correlation was also reported between minerality and residual sugars [32], similar to our results that demonstrated a significative negative correlation between *sapidity* and *sweetness* (Figure 3B). Furthermore, results showed that *sweetness* was positively correlated in Timorasso wines with fructose and glucose contents, as well as with acetic acid (Figure 2). One of the hypotheses for the latter phenomenon is that higher contents of acetic acid (and volatile acidity) are found in wines with difficulties completing alcoholic fermentation. Also, our study revealed that residual sugars were correlated with *smoothness* (Figure 2), which, in turn, was a significant driver of *In-Mouth typicity* (*r* = 0.465, *p* < 0.01, Figure 3B).

Concerning the Correspondence Analysis (CA, Figure 3A), Dimensions 1 and 2 accounted for 31.68% and 14.22% of the explained variance, respectively, and the results agreed with the tendencies described above. The first Dimension correlated with *In-Mouth typicity* (*r* = −0.3676, *p* = 0.0419): on the negative side of the plot were the descriptors *sapidity*, *acidity*, and *minerality*, and on the opposite side was *sweetness*. For the second Dimension, the correlation with *In-Mouth typicity* was *r* = 0.5958 (*p* < 0.001). This Dimension also allowed discrimination between the vintages 2019 and 2016: the 2019 wines (on the upper side of the graph) were associated with *smoothness*, *texture*, and *body*, whereas the older 2016 vintage samples (on the opposite side) had more *bitterness*.

### 3.3. Aroma Descriptors and Their Correlation with Aroma Typicity

The overall *Liking* of Timorasso wines was highly correlated with *Aroma typicity* (*r* = 0.857, *p* < 0.001, Table S5). The citation frequencies of individual attributes and their “Categories” are presented in Table 3. The most frequently mentioned category was “Kerosene” (CF: 27.9%), which comprises *hydrocarbon* (18.7%), *kerosene/TDN* (6.9%), and *petroleum/diesel* (3.2%) descriptors. This category correlated well with *Aroma typicity* (*r* = 0.7771, *p* < 0.001), and it was also highly significant in discriminating between the samples (*p* < 0.001), meaning that not all the wines showed these distinct *kerosene* notes. The second most frequently cited category was “White flowers” (15.5%), with the attributes *white flowers* (8.1%) and *acacia flowers* (5.3%). This category significantly differentiated the samples (*p* < 0.001), although it was not correlated with the *Aroma typicity* of Timorasso wines.

The third most often mentioned category was “Yellow pulp fruits” (14.8%), with the following descriptors mentioned: *peach* (4.5%), *apricot* (4.8%), and *yellow plum* (3.5%). There were also a few other categories related to fruitiness with relatively high citation frequency, about 10–12%, such as “Citrus” (11.1%), “Tropical fruits” (10.6%), “White pulp fruits” (9.8%), etc. Within these categories, the important individual descriptors were *pear* (5.3%), *apricot* (4.8%), *peach* (4.5%), *citrus* (4.0%), and *apple* (4.0%). All of these categories were able to discriminate between the wine samples but did not correlate with *Aroma typicity*. The descriptors listed in this paragraph are often associated with ethyl and acetate esters typically found in high concentrations in wines of various varieties and are derived from yeast metabolism during alcoholic fermentation. This explains the lack of significant correlations between these fruit categories and *Aroma typicity* (Table 3); nevertheless, the experts gave a positive assessment of these fruit attributes.

Besides “Kerosene”-related individual descriptors, the most mentioned non-fruity/non-floral ones were *balsamic* (7.1%), *almond* (6.6%), and *hay/straw* (6.1%). As for the first one, it was commonly present in the samples, and its category “Balsamic” (10.8%) was positively correlated with the sensory identity (*Aroma typicity*) of Timorasso wines (*r* = 0.4913, *p* = 0.0050). *The almond* descriptor is listed under the category “Nuts” (11.8%), while *hay/straw* belongs to the category “Hay/herbs” (6.1%). These two categories did not discriminate the samples, and they were not related to the *Aroma typicity* of Timorasso wines (no significant correlations). In addition to the abovementioned descriptors, some notes of wine evolution can be represented in Timorasso wines by aromas in the categories “Dried fruits” (9.2%) and/or “Honey” (11.3%), which were perceived in some samples.

Regarding reduction/oxidation aroma descriptors, it is worth mentioning *sulphur* (from the category “Reduced”) with a citation frequency of 3.9%. The presence of sulphur compounds in wine in higher concentrations may lead to certain unpleasant reductive notes. On the other hand, some sulphur compounds such as methanethiol, benzenemethanethiol, and analogs, may be related to a positive category of descriptors called “Empyreumatic” [35,36], which can be characterized by *flint*, *burnt rubber*, and *mineral* hints. In our study, the “Empyreumatic” category and flint descriptor had citation frequencies of 5.5% and 3.2%, respectively. Moreover, the “Empyreumatic” category was significantly implicated in *Aroma typicity* (correlation: *r* = 0.4343, *p* = 0.0146). As for oxidation-related wine aromas, the “Light oxidation” category was mentioned with a citation frequency of 12.1% and included *marsala-like* and *brandy-like* individual descriptors. “Pungent oxidation” (9.0%) was a category giving a rather negative sensory impression with the prevailing individual descriptor *oxidized* (6.0%) and, to a lesser extent, *acetic acid* and *ethyl acetate*. Both oxidation-related categories were highly significant in the negative implication in *Aroma typicity* (*r* = −0.5683 and *r* = −0.5118 for “Pungent oxidation” and “Light oxidation”, respectively) and in discriminating the wine samples, meaning that the oxidation fault affected only a minor part of the Timorasso wines investigated.

### 3.4. Influence of Wine Aging on Aroma Sensory Descriptors

The first stage of the results visualization was conducted using all individual descriptors reported with a citation frequency higher than 1% (Figure S3). Obviously, due to the large number of descriptors, the explained variance by the first two axes was limited: Dimension 1 accounted for 17.08% of the explained variance, and Dimension 2 for 12.77%. Dimension 1 was negatively correlated with *Aroma typicity* (*r* = −0.3929; *p* = 0.0288) and allowed discrimination of one of the oldest (2016) vintages from the youngest one (2021). Thus, it was possible to distinguish the samples from the 2021 vintage (*r* = −0.7497, *p* = 0.0012) on the left side of the plot, next to the descriptors *linden flowers*, *green*, *lemon*, *pear*, *banana*, and *citrus*. Conversely, older samples from the 2016 vintage could be found on the right side (*r* = 0.8261, *p* = 0.0171) and were characterized by *oxidized*, as well as *jam*, *brandy-like*, and *candied fruits* descriptors. These wines were considered adequate in the ratings evaluation (Table S4); nevertheless, the smallest number of observations of the oldest vintages (2015–2016) should be considered in the overall evaluation. As for Dimension 2, it negatively correlated with *Aroma typicity* (*r* = −0.6103, *p* = 0.0003) and allowed to separate samples of the 2021 vintage (*r* = 0.4655, *p* = 0.0102) from the wines of the 2017 vintage (*r* = −0.4369, *p* = 0.0438). The positive side of Dimension 2 was related to the descriptors *lemon*, *linden flowers*, *pineapple*, and *lime*, whereas the other side was characterized by *flint* and *kerosene* hints, *mint*, *vanilla*, *resin/pine*, and *clove*, which are the most correlated with the *Aroma typicity* of Timorasso wines.

**Figure 4:**
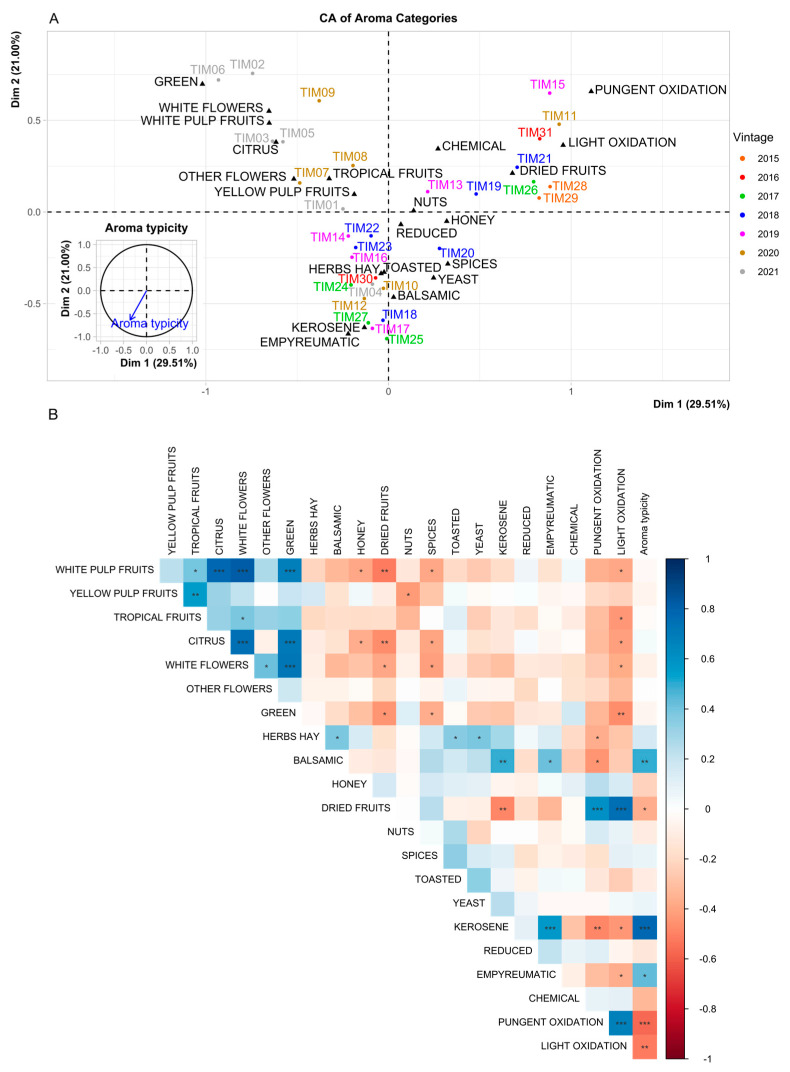
Correspondence analysis of the aroma descriptors (**A**) and correlation plot of descriptors and *Aroma typicity* (**B**). Sign.: *, **, and ***, indicate significant correlation at *p* < 0.05, 0.01, 0.001.

When the individual descriptors were grouped into “Categories” (Figure 4), the results agreed with Figure S3, but with the increased power of the variance explanation of 50.51% from the first two dimensions: 29.51% for Dimension 1 and 21.00% for Dimension 2. Dimension 1 was negatively correlated with Timorasso wines’ *Aroma typicity* (*r* = −0.3567, *p* = 0.0489). The main contributing categories on the right side were “Pungent oxidation” and “Light oxidation”, followed by the categories of descriptors associated with wine evolution, such as “Dried fruits”, “Spices”, and “Honey”. The ”Dried fruits” category was highly correlated with the two oxidation-related categories (“Pungent oxidation” and “Light oxidation”, *r* = 0.607 and *r* = 0.751, respectively, both *p* < 0.001, Figure 4B), whereas on the opposite side of the graph, “Green”, “White flowers”, “White pulp fruits”, and “Citrus” characterized Dimension 1. These categories were correlated with each other to different extents, which probably characterize young wines (Figure 4B). In fact, Dimension 1 allowed to separate well the older and newer vintages, with the 2021 vintage being significantly different from the 2015 vintage (*r* = −0.6565, *p* = 0.0013 and *r* = 0.7184, *p* = 0.0235, respectively). Dimension 2 was also negatively correlated with *Aroma typicity* (*r* = −0.638 and *p* = 0.0001). In this case, “Empyreumatic”, “Kerosene”, and “Balsamic” (bottom of the graph, Figure 4A) had a higher correlation with Dimension 2 and were also positively correlated with each other, showing a concomitant increase (Figure 4B). Also, at the bottom of the graph were wines from the 2017 vintage, separated by Dimension 2 (*r* = −0.3881, *p* = 0.0426). As shown in the graph of supplementary variables (Figure 4), *Aroma typicity* is well represented by this quadrant.

## 4. Discussion

The 31 wines studied, made from ‘Timorasso’ grapes, may be divided into three main groups, depending (mainly, but not only) on the wine age with (1) fresh fruity and floral notes; (2) reductive aging notes (e.g., *kerosene*, *flint*); and (3) evolved/oxidative (more or less impacting) aging notes. The experts evaluated the typicity of Timorasso wines in relation to the categories “Empyreumatic”, “Kerosene”, and “Balsamic”, and the corresponding aromas can reach noticeable levels in the wines after 5–6 years of aging (e.g., 2017 vintage samples tasted in 2023, Figure 4). This characteristic is shared by other wines from international varieties, such as certain Chardonnay and Sauvignon blanc wines showing “Empyreumatic” notes [35,36,37], as well as certain Riesling wines for the “Kerosene” and “Balsamic” categories [38,39,40,41,42].

Recent analyses reported that Timorasso grapes have a low concentration of terpenes, which usually contribute to the floral/fruity aromas in many young wines [14,16]. The lack of fruity notes and the presence of reductive aromas, here reported as “Empyreumatic” and “Reduced” categories, have been found to be positively correlated with the *minerality* in Chardonnay and Sauvignon blanc wines [32,36]. However, preliminary chemical analyses revealed a higher presence of other compounds in Timorasso, C_13_-norisoprenoids, which provide wine aromas that may develop and intensify over time [14,16]. This class of compounds has been involved in the *kerosene*-type of notes, with TDN being the main chemical substance responsible for these aromas [38,39,40,41,42]. Another C_13_-norisoprenoid, vitispirane, may contribute to *camphor* and *eucalyptus* notes but also has a higher sensory threshold [39]. The number of aroma compounds (including TDN and vitispirane) can increase during wine aging [42] and can be affected by the closure type. In our case, if the closure type (Table S1) is used as a factor in the CA (Figure 3), Dimension 1 separates well the wines sealed with synthetic stoppers (*r* = 0.4060, *p* = 0.0020) from the wines with screw caps (*r* = −0.4800, *p* = 0.0151). This is relevant since several studies on Riesling showed that the plastic stoppers can scalp TDN considerably from wine, while screw caps were found to be the best closures for preserving it [42]. However, it must be noted that this study was not designed on this factor, so not all types of closures are present in every vintage.

The 2016 wine samples, along with some other Timorasso wines from different vintages, were characterized by descriptors of oxidative aging, such as “Honey”, “Dried fruits”, “Light oxidation”, “Nuts”, and “Pungent oxidation”. Light oxidative attributes are not necessarily negative and are less related to *Aroma typicity* ratings. Using oak for wine aging can increase the perception of corresponding aromas such as *toasted*, *dried fruits*, and *caramel* [14]. Oak aging is allowed according to the designation regulations; however, it is not a common practice in this area [15]. The situation is similar to the prolonged skin contact technique, which is not usual in wine production, but certain orange-type Timorasso wines can be found. This technique can provide fruit notes, as was the case with the relatively young wine TIM11 (vintage 2020), and was found to increase the *petrol* notes in other varieties [43,44]. Thus, variations in vinification strategies can be a stronger factor affecting the wine aroma composition than vintage or wine age.

## 5. Conclusions

This study is the first stage in our research on wines made from the rediscovered grape variety ‘Timorasso’. It provides a broad characterization of 31 wine samples from the 2015–2021 vintages and the selection of typical samples. The initial analysis focused on basic physicochemical parameters and sensory descriptors. The sensory analysis was conducted with wine professionals, who were also asked to evaluate the typicity of the wines.

Timorasso wines, produced with different aging times and winemaking methods, were generally described as high in *acidity*, *sapidity*, and *minerality* in mouth traits that correlated with their typicity and organic acids content. Aromatic descriptors evolve greatly during aging depending on the winemaking style. Notably, the “Kerosene“, “Balsamic”, and “Empyreumatic” categories were most correlated with the wine typicity, particularly in wines with 5–6 years of aging. In contrast, the young wines were characterized by fruity and floral notes. A subset of wines produced in a more oxidative winemaking style or with longer aging showed more *toasted* and *dried fruits* notes.

Future research on the typicity of Timorasso wines may involve the determination of volatile organic compounds (including varietal aromas) in selected Timorasso wines using instrumental analysis and their correlation with sensory characteristics. Further studies can also be aimed at the exploration of vinification and aging factors to provide technological awareness on how to manage sensory markers related to the typicity of Timorasso-based wines.

## Figures and Tables

**Table 1:** Color parameters and total polyphenol content of the Timorasso wines analyzed.

	Sample	L*	a*	b*	C	H°	Abs 420 nm (10 mm, O.P.)	Visual Color (RGB)	Total Polyphenols (mg/L EC)
2021	TIM01	97.7 ± 0.0	−2.23 ± 0.01	13.49 ± 0.08	13.67 ± 0.08	99.40 ± 0.03	0.206 ± 0.001		953.1 ± 2.8
	TIM02	98.2 ± 0.0	−1.33 ± 0.02	9.30 ± 0.02	9.40 ± 0.02	98.14 ± 0.12	0.138 ± 0.001		731.1 ± 1.1
	TIM03	98.8 ± 0.2	−1.03 ± 0.01	6.35 ± 0.10	6.43 ± 0.10	99.21 ± 0.21	0.092 ± 0.003		603.2 ± 0.0
	TIM04	98.8 ± 0.0	−0.91 ± 0.01	6.25 ± 0.04	6.31 ± 0.04	98.27 ± 0.12	0.088 ± 0.000		506.8 ± 3.9
	TIM05	98.7 ± 0.1	−1.34 ± 0.00	7.75 ± 0.03	7.87 ± 0.02	99.83 ± 0.06	0.112 ± 0.002		857.9 ± 3.9
	TIM06	98.6 ± 0.0	−1.38 ± 0.02	8.28 ± 0.03	8.40 ± 0.03	99.45 ± 0.09	0.120 ± 0.001		655.1 ± 2.8
	**2021**	**98.5** ± **0.4**	**−1.37** ± **0.46**	**8.57** ± **2.68**	**8.68** ± **2.71**	**99.05** ± **0.69**	**0.126** ± **0.043**		**717.9** ± **165.4 A**
2020	TIM07	98.2 ± 0.0	−1.90 ± 0.01	11.12 ± 0.00	11.28 ± 0.00	99.71 ± 0.04	0.163 ± 0.000		769.4 ± 4.4
	TIM08	98.6 ± 0.0	−1.17 ± 0.01	7.73 ± 0.05	7.82 ± 0.05	98.62 ± 0.02	0.111 ± 0.001		708.1 ± 3.9
	TIM09	98.0 ± 0.0	−1.16 ± 0.02	9.51 ± 0.06	9.58 ± 0.06	96.95 ± 0.07	0.141 ± 0.000		730.4 ± 3.3
	TIM10	97.2 ± 0.0	−1.77 ± 0.01	15.25 ± 0.02	15.36 ± 0.02	96.62 ± 0.03	0.218 ± 0.000		1108.8 ± 24.3
	TIM11	91.4 ± 0.0	1.69 ± 0.01	30.26 ± 0.00	30.31 ± 0.00	86.81 ± 0.03	0.437 ± 0.000		1417.8 ± 5.5
	TIM12	97.1 ± 0.2	−2.13 ± 0.02	13.24 ± 0.05	13.40 ± 0.05	99.13 ± 0.11	0.204 ± 0.003		862.6 ± 8.3
	**2020**	**96.7 ± 2.7**	**−1.07** ± **1.41**	**14.52** ± **8.16**	**14.63** ± **8.13**	**96.31** ± **4.81**	**0.212** ± **0.117**		**932.8** ± **279.0 A**
2019	TIM13	96.8 ± 0.0	−1.38 ± 0.01	15.46 ± 0.02	15.52 ± 0.01	95.11 ± 0.03	0.222 ± 0.000		826.3 ± 3.3
	TIM14	98.0 ± 0.0	−1.74 ± 0.02	11.23 ± 0.01	11.37 ± 0.01	98.82 ± 0.07	0.168 ± 0.000		823.2 ± 5.5
	TIM15	97.2 ± 0.0	−1.79 ± 0.04	14.24 ± 0.05	14.36 ± 0.05	97.17 ± 0.12	0.213 ± 0.001		657.0 ± 2.2
	TIM16	98.0 ± 0.0	−1.67 ± 0.00	11.22 ± 0.04	11.35 ± 0.04	98.44 ± 0.02	0.165 ± 0.001		741.3 ± 1.1
	TIM17	98.1 ± 0.0	−1.76 ± 0.01	11.73 ± 0.01	11.86 ± 0.02	98.54 ± 0.02	0.169 ± 0.000		721.4 ± 1.7
	**2019**	**97.6** ± **0.6**	**−1.67** ± **0.17**	**12.78** ± **1.95**	**12.89** ± **1.93**	**97.62** ± **1.54**	**0.187** ± **0.028**		**753.8** ± **71.9 A**
2018	TIM18	97.4 ± 0.1	−1.72 ± 0.01	13.51 ± 0.01	13.62 ± 0.01	97.25 ± 0.04	0.201 ± 0.010		723.3 ± 3.3
	TIM19	93.4 ± 0.1	−1.55 ± 0.03	26.99 ± 0.06	27.04 ± 0.06	93.28 ± 0.08	0.423 ± 0.002		1152.9 ± 0.6
	TIM20	98.6 ± 0.0	−1.49 ± 0.00	8.69 ± 0.00	8.82 ± 0.00	99.75 ± 0.01	0.124 ± 0.000		742.8 ± 1.1
	TIM21	96.6 ± 0.0	−0.92 ± 0.03	14.67 ± 0.02	14.70 ± 0.02	93.58 ± 0.11	0.212 ± 0.001		689.4 ± 1.7
	TIM22	95.5 ± 0.0	−2.65 ± 0.00	23.73 ± 0.02	23.87 ± 0.02	96.36 ± 0.01	0.371 ± 0.001		1079.5 ± 2.8
	TIM23	98.2 ± 0.0	−1.85 ± 0.00	10.98 ± 0.02	11.13 ± 0.02	99.56 ± 0.04	0.155 ± 0.000		838.0 ± 2.2
	**2018**	**96.6** ± **1.9**	**−1.69** ± **0.56**	**16.69** ± **7.30**	**16.79** ± **7.28**	**96.57** ± **2.80**	**0.252** ± **0.120**		**884.6** ± **191.3 A**
2017	TIM24	98.9 ± 0.0	−1.81 ± 0.01	8.67 ± 0.02	8.85 ± 0.02	101.78 ± 0.07	0.126 ± 0.000		664.0 ± 5.5
	TIM25	98.8 ± 0.1	−1.68 ± 0.01	8.23 ± 0.01	8.40 ± 0.01	101.51 ± 0.05	0.120 ± 0.001		639.1 ± 1.1
	TIM26	96.4 ± 0.1	−0.75 ± 0.02	13.37 ± 0.04	13.39 ± 0.04	93.20 ± 0.08	0.196 ± 0.001		587.9 ± 1.7
	TIM27	95.9 ± 0.0	−1.35 ± 0.01	17.56 ± 0.00	17.62 ± 0.00	94.39 ± 0.04	0.256 ± 0.001		699.1 ± 0.0
	**2017**	**97.5** ± **1.6**	**−1.39** ± **0.47**	**11.96** ± **4.40**	**12.06** ± **4.33**	**97.72** ± **4.56**	**0.174** ± **0.059**		**647.5** ± **46.8 A**
2016	TIM28	96.7 ± 0.1	−0.80 ± 0.06	12.41 ± 0.02	12.44 ± 0.02	93.71 ± 0.28	0.186 ± 0.001		499.0 ± 1.7
	TIM29	96.1 ± 0.0	−0.69 ± 0.00	14.91 ± 0.00	14.93 ± 0.00	92.63 ± 0.01	0.226 ± 0.000		566.5 ± 3.3
	**2016**	**96.4** ± **0.3**	**−0.75** ± **0.08**	**13.66** ± **1.77**	**13.68** ± **1.76**	**93.17** ± **0.76**	**0.206** ± **0.023**		**532.7** ± **47.7 A**
2015	TIM30	98.3 ± 0.2	−2.63 ± 0.01	12.73 ± 0.06	13.00 ± 0.06	101.68 ± 0.03	0.192 ± 0.003		1188.8 ± 2.8
	TIM31	95.5 ± 0.0	−1.27 ± 0.00	18.53 ± 0.02	18.57 ± 0.02	93.91 ± 0.01	0.273 ± 0.000		715.9 ± 0.6
	**2015**	**96.9** ± **1.9**	**−1.95** ± **0.97**	**15.63** ± **4.10**	**15.79** ± **3.94**	**97.79** ± **5.49**	**0.233** ± **0.047**		**952.3** ± **334.4**
	**All**	**97.3** ± **1.7**	**−1.42** ± **0.76**	**13.14** ± **5.62**	**13.25** ± **5.61**	**97.19** ± **3.28**	**0.194** ± **0.085**		**789.0** ± **210.4 A**
**Sign. Sample**		**	*	**	**	**	**		***
**Sign. Vintage**		ns	ns	ns	ns	ns	ns		*

Data are expressed as average value ± standard deviation of two measurements for “sample”. For “vintage”, these are average values of the samples ± standard deviations. Sign.: *, **, ***, and “ns” indicate significant differences at *p* < 0.05, 0.01, 0.001, and not significant, respectively, according to Kruskal–Wallis test. Uppercase letter indicates the difference for Dunn test (*p* < 0.05) for the factor “vintage”. Visual Color estimation was obtained by conversion of L*, a*, and b* values to RGB values.

**Table 2:** Citation frequency (CF) of *In-Mouth individual descriptors*, their significance in wines discrimination, and correlation with *In-Mouth typicity*.

*Individual Descriptors*	*Individual* *Descriptor* CF (%)	Sign. *Individual* *Descriptors* *	*r* *In-Mouth Typicity*	Sign. Correlation
*Acidity*	32.9	**0.0000**	0.1145	0.5395
*Bitterness*	11.9	**0.0192**	−0.2213	0.2316
*Sweetness*	13.4	**0.0001**	−0.3531	0.0514
*Astringency*	7.1	0.2754	−0.0025	0.9893
*Body*	12.4	0.3190	0.2784	0.1294
*Texture*	8.4	0.4850	0.3282	0.0714
*Warmth Alcohol*	12.7	0.7184	−0.1567	0.3998
***Sapidity*** **^#^**	25.5	**0.0003**	**0.3892**	**0.0305**
***Minerality***	17.4	**0.0579**	**0.6040**	**0.0003**
***Smoothness***	11.5	0.6549	**0.4654**	**0.0083**

Data are expressed as frequency for each *individual descriptor* calculated as percentage (%) = number of *individual descriptor* occurrence/(number of judges x number of wine samples), with the latter corresponding judges = 20 and samples = 31. * Significant difference for *individual descriptors* were calculated according to the Cochran’s Q test. Pearson correlation (*r*) statistical significance is calculated according to *t*-test. Descriptors in bold are significantly discriminating the wines according to Cochran’s Q test (*p* < 0.10) or significantly correlated with *In-Mouth typicity* (*p* < 0.05). ^#^ Sapidity/Savoriness/Saltiness.

**Table 3:** Citation frequency (CF) of *individual descriptors* and corresponding “Categories”, their significance in wines discrimination, and correlation with *Aroma typicity*.

“Categories”	*Individual* *Descriptors*	*Individual* *Descriptor* CF (%)	Sign. *Individual* *Descriptor* *	“Category” CF (%)	Sign. Category *	*r Aroma* *Typicity*	Sign. Correlation
“White pulp fruits”	*Apple*	4.0	**0.0250**	9.8	**0.0001**	−0.0338	0.8566
	*Pear*	5.3	**0.0020**				
	*White pulp fruits*	1.6	0.5930				
“Yellow pulp fruits”	*Peach*	4.5	0.4370	14.8	**0.0004**	−0.0578	0.7575
	*Apricot*	4.8	0.1690				
	*Yellow plum*	3.5	0.7010				
	*Yellow pulp fruits*	2.6	0.3770				
“Tropical fruits”	*Banana*	1.9	**0.0016**	10.6	**0.0116**	−0.0185	0.9212
	*Mango*	2.6	0.1920				
	*Melon*	0.8	0.6570				
	*Passion fruit*	1.5	0.1810				
	*Pineapple*	2.1	**0.0038**				
	*Tropical fruits*	3.7	0.1740				
“Citrus”	*Lemon*	2.4	**0.0213**	11.1	**0.0000**	0.0329	0.8604
	*Lime*	1.6	0.2868				
	*Grapefruit*	3.2	0.6896				
	*Citrus*	4.0	0.1924				
“White flowers”	*Acacia flowers*	5.3	**0.0024**	15.5	**0.0000**	−0.0761	0.6839
	*Linden flowers*	2.3	**0.0003**				
	*Elder flowers*	0.6	1.0000				
	*Orange blossom*	0.3	1.0000				
	*Jasmine*	1.5	0.1810				
	*White flowers*	8.1	**0.0000**				
“Other flowers”	*Rose*	1.1	0.0593	2.7	**0.0063**	0.0082	0.9650
	*Violet*	1.0	0.1906				
	*Lavander*	0.5	0.5367				
“Green”	*Green (fresh grass)*	2.4	0.1448	2.4	0.1448	−0.0444	0.8127
“Herbs/hay”	*Tea*	2.6	**0.0007**	10.8	0.0559	0.1572	0.3983
	*Aromatic herbs*	2.3	0.2377				
	*Medicinal herbs*	0.5	**0.0170**				
	*Hay/straw*	6.1	0.6523				
	*Tobacco*	1	0.7113				
	*Vegetal*	1.3	0.7720				
“Balsamic”	*Resin/pine*	1.3	0.7720	10.8	0.1051	**0.4913**	**0.0050**
	*Mint*	1.1	0.7505				
	*Camphor*	0.5	0.5704				
	*Eucalyptus*	1.1	0.3015				
	*Balsamic*	7.1	0.1967				
“Honey”	*Camomile*	1.9	0.7180	11.3	**0.0437**	−0.2252	0.2232
	*Broom*	0.6	0.5741				
	*Honey*	9.0	0.1185				
“Dried fruits”	*Candied fruits*	2.3	**0.0106**	9.2	0.1261	**−0.3610**	**0.0460**
	*Jam*	1.0	0.1906				
	*Raisin*	0.8	0.6383				
	*Fig*	5.0	0.0636				
	*Dried fruits*	2.4	0.2479				
“Nuts”	*Amaretto*	0.5	0.5704	11.8	0.5412	−0.1005	0.5908
	*Hazelnut*	2.7	0.4079				
	*Almond*	6.6	0.6188				
	*Coconut*	1.1	0.7615				
	*Walnut*	0.6	0.5992				
	*Nuts*	1.8	0.5986				
“Spices”	*Licorice*	2.4	0.2357	10.8	**0.0030**	0.0759	0.6849
	*Vanilla*	2.7	**0.0005**				
	*Nutmeg*	2.6	0.3969				
	*Anise*	0.6	0.0648				
	*White pepper*	0.5	1.0000				
	*Cinnamon*	0.5	0.5368				
	*Clove*	1.5	0.5833				
	*Spices*	1.8	0.6526				
“Toasted”	*Caramel*	1.8	**0.0490**	5.3	**0.0000**	−0.0949	0.6115
	*Coffee*	0.3	0.5176				
	*Smoke*	1.6	0.0875				
	*Toasted*	1.8	0.3837				
“Yeast”	*Bread crust*	1.5	0.6751	3.2	0.5603	0.0698	0.7091
	*Butter*	0.8	0.5121				
	*Yeast*	2.3	0.5555				
“Kerosene”	*Kerosene/TDN*	6.9	**0.0001**	27.9	**0.0000**	**0.7771**	**0.0000**
	*Hydrocarbon*	18.7	**0.0000**				
	*Petroleum/diesel*	3.2	0.1417				
“Reduced”	*Cooked cabbage*	1.0	0.6668	4.7	0.2626	−0.1267	0.4971
	*Sulphur*	3.9	**0.0724**				
	*Hydrogen sulphide*	0.8	0.6571				
	*Reduced*	0.6	0.2047				
“Empyreumatic”	*Flint*	3.2	**0.0952**	5.5	**0.01317**	**0.4343**	**0.0146**
	*Burnt rubber*	0.6	0.5991				
	*Mineral*	1.6	**0.0923**				
“Chemical”	*Solvent*	1.5	**0.0588**	3.7	**0.0353**	−0.3276	0.0720
	*Ethanol*	0.8	0.6383				
	*Plastic*	2.1	0.1193				
	*Chemical*	0.3	0.5176				
“Pungent oxidation”	*Acetic acid*	1.3	0.3377	9.0	**0.0000**	**−0.5683**	**0.0009**
	*Acetaldehyde*	2.1	**0.0126**				
	*Ethyl acetate*	1.0	0.7250				
	*Oxidized*	6.0	**0.0000**				
	*Pungent*	0.5	**0.0170**				
“Light oxidation”	*Brandy-like*	4.0	**0.0031**	12.1	**0.0000**	**−0.5118**	**0.0032**
	*Marsala-like*	5.3	**0.0009**				
	*Light oxidized*	3.9	**0.0054**				

Data are expressed as frequency (CF) for each *individual descriptor*, and for “Category”, they were calculated as percentage (%) = number of *individual descriptor* or “Category” occurrence/(number of judges ×number of wine samples), with the latter corresponding to judges = 20 and samples = 31. * Significant differences for each *individual descriptor* and “Category” were calculated according to Cochran’s Q test. Pearson correlation (*r*) statistical significance is calculated according to *t*-test. Values in bold indicate significant *individual descriptors*/“Categories” according to Cochran’s Q test (*p* < 0.10) or significantly correlated with Aroma typicity (*p* < 0.05). Values highlighted in grey show CF < 1%.

## Data Availability

The original contributions presented in the study are included in the article; further inquiries will be made available upon reasonable request and in line with the consent agreed with participants by contacting the corresponding authors.

## Supplementary Materials

The following supporting information can be downloaded at https://www.mdpi.com/article/10.3390/foods14040591/s1, Table S1: Timorasso wines analyzed in the study; Table S2: Sensory Descriptors (GROUP, “Category”, *individual descriptors*); Table S3: Basic physicochemical parameters of the analyzed wines; Table S4: Sensory results for 0–10 continuous scale evaluation of color, typicity, and liking; Table S5: Pearson Correlation among color, typicity and liking scales; Figure S1: Example of the proposed tasting sheet; Figure S2: Pearson correlation plot of visual color characteristics and colorimetric instrumental measurements; Figure S3: Correspondence Analysis (CA) of the individual descriptors (frequency > 1%).
