# Supplementary material for: Exploring the Sensory Typicity of Timorasso Wines: Physicochemical and Sensory Characteristics of Seven Consecutive Vintages
Source: Foods. 2025 Feb 11;14(4):591. doi: 10.3390/foods14040591 (PMC11854514; doi:10.3390/foods14040591)

Table S1. Timorasso wines analysed in the study.

Vintage	Sample Code	Closure Type	Designation
2021	TIM01	Agglomerated cork	Colli Tortonesi DOC Timorasso
	TIM02	Synthetic stopper	Colli Tortonesi DOC Timorasso
	TIM03	Natural cork	Colli Tortonesi DOC Timorasso
	TIM04	Agglomerated cork	Colli Tortonesi DOC Timorasso
2020	TIM05	Synthetic stopper	Colli Tortonesi DOC Timorasso
	TIM06	Synthetic stopper	Colli Tortonesi DOC Timorasso
	TIM07	Agglomerated cork	Colli Tortonesi DOC Timorasso
	TIM08	Agglomerated cork	Colli Tortonesi DOC Timorasso
	TIM09	Synthetic stopper	Colli Tortonesi DOC Timorasso
	TIM10	Agglomerated cork	Colli Tortonesi DOC Timorasso
2019	TIM11	Agglomerated cork	Colli Tortonesi DOC Timorasso
	TIM12	Screw cap	Colli Tortonesi DOC Timorasso
	TIM13	Synthetic stopper	Colli Tortonesi DOC Timorasso
	TIM14	Synthetic stopper	Colli Tortonesi DOC Timorasso Riserva
	TIM15	Synthetic stopper	Colli Tortonesi DOC Timorasso
2018	TIM16	Natural cork	Colli Tortonesi DOC Timorasso
	TIM17	Agglomerated cork	Colli Tortonesi DOC Timorasso
	TIM18	Screw cap	Colli Tortonesi DOC Timorasso
	TIM19	Natural cork	Colli Tortonesi DOC Timorasso
	TIM20	Synthetic stopper	Colli Tortonesi DOC Timorasso
	TIM21	Agglomerated cork	Colli Tortonesi DOC Timorasso Riserva
2017	TIM22	Natural cork	Colli Tortonesi DOC Timorasso
	TIM23	Agglomerated cork	Colli Tortonesi DOC Timorasso
	TIM24	Agglomerated cork	Vino bianco (White wine)
	TIM25	Agglomerated cork	Colli Tortonesi DOC Timorasso
	TIM26	Synthetic stopper	Colli Tortonesi DOC Timorasso
	TIM27	Screw cap	Vino bianco (White wine)
2016	TIM28	Natural cork	Vino bianco (White wine)
	TIM29	Synthetic stopper	Colli Tortonesi DOC Timorasso
2015	TIM30	Agglomerated cork	Colli Tortonesi Terre di Libarna DOC
	TIM31	Natural cork	Vino bianco (White wine)

Table S2. Sensory Descriptors (GROUP, "Category", *individual descriptors*). The descriptors are reported in Italian as proposed to the panel and their translation in English adopted for this study.

**AROMA (Orthonasal + Retronasal)**	
**FRUTTA**	**FRUITY**
**"Frutta polpa bianca":** *Mela, Pera, Mela verde, Uva spina*	**"White pulp fruits"**: *Apple, Pear, Green apple, Gooseberry*
**"Frutta polpa gialla":** *Pesca, Albicocca, Prugna gialla*	**"Yellow pulp fruits":** *Peach, Apricot, Yellow Plum*
**"Frutta tropicale":** *Banana, Mango, Melone, Frutto della passione, Ananas, Litchi, Papaya*	**"Tropical fruits:"** *Banana, Mango, Melon, Passion fruit, Pineapple, Lychee, Papaya*
**"Agrumi":** *Limone, Lime, Pompelmo, Arancio*	**"Citrus":** *Lemon, Lime, Grapefruit, Orange*
**FIORI**	**FLORAL**
**"Fiori bianchi":** *Fiori di acacia, Fiori di tiglio, Fiori di sambuco, Fiori d'arancio, Gelsomino*	**"White flowers":** *Acacia flowers, Linden flowers, Elder flowers, Orange blossom, Jasmine*
**"Fiori colorati":** *Rosa, Violetta, Lavanda, Geranio*	**"Other flowers":** *Rose, Violet, Lavender, Geranium*
**VEGETALE**	**VEGETAL**
**"Erbe fresche (verde)":** *Erba tagliata, Peperone*	**"Fresh (green)":** *Fresh grass, Bell pepper*
**"Erbe essiccate":** *Foglie di Tè, Erbe aromatiche, Erbe medicinali, Fieno/Paglia, Tabacco*	**"Hay/herbs":** *Tea, Aromatic herbs, Medicinal herbs, Hay/straw, Tobacco*
**"Balsamico":** *Resina/pino, Menta, Canfora, Eucalipto*	**"Balsamic":** *Resin/pine, Mint, Camphor, Eucalyptus*
**AFFINAMENTO/EVOLUZIONE**	**AGING/EVOLUTION**
**"Miele":** *Camomilla, Ginestra, Melassa*	**"Honey":** *Camomille, Broom, Molasses*
**"Frutta disidratata":** *Frutta candita, Marmellata, Uvetta, Fichi, Prugna, Albicocca*	**"Dried fruits":** *Candied fruits, Jam, Raisin, Fig, Plum, Apricot*
**"Frutta secca":** *Amaretto, Nocciola, Mandorla, Cocco, Noce*	**"Nuts"**: *Amaretto, Hazelnut, Almond, Coconut, Walnut,*
**"Spezie":** *Liquirizia, Vaniglia, Noce moscata, Anice, Pepe bianco, Cannella, Chiodi di garofano*	**"Spices":** *Licorice, Vanilla, Nutmeg, Anise, White Pepper, Cinnamon, Cloves*
**"Tostato":** *Caramello, Caffè, Fumo, Pane Tostato*	**"Toasted":** *Caramel, Coffee, Smoke, Toasted Bread*
**MICROBIOLOGICO**	**MICROBIOLOGICAL**
**"Lievito":** *Burro, Crosta di pane*	**"Yeast":** *Butter, Bread Crust*
**CHIMICO**	**CHEMICAL**
**"Petrolio":** *Cherosene/TDN, Idrocarburo, Petrolio/Diesel, Catrame*	**"Kerosene":** *Kerosene/TDN, Hydrocarbon, Petroleum/Diesel, Tar*
**"Ridotto":** *Cavolo cotto, Sulfureo, Idrogeno Solforato, Anidride solforosa*	**"Reduced":** *Cooked Cabbage, Sulphur, Hydrogen Sulphide, Sulfur dioxide*
**"Empireumatico":** *Pietra focaia, Gomma bruciata, Minerale*	**"Empyreumatic":** *Flint, Burnt rubber, Mineral*
**"Chimico":** *Solvente, Etanolo, Plastic*	**"Chemical":** *Solvent, Ethanol, Plastic*
**OSSIDATO**	**OXIDIZED**
**"Pungente":** *Acido acetico, Acetaldeide, Acetato di etile*	**"Pungent oxidation":** *Acetic acid, Acetaldehyde, Ethyl acetate*
**"Leggera ossidazione":** *Marsalato, Simil-Brandy,*	**"Light oxidation":** *Marsala-like, Brandy-like*
**SENSAZIONI IN BOCCA**	**IN-MOUTH**
**"Gusti":** *Acidità, Amaro, Dolce*	**"Taste":** *Acidity, Bitterness, Sweetness*
**"Altri":** *Astringenza, Corpo, Struttura, Calore-Alcol, Sapidità, Mineralità, Morbidezza*	**"Others":** *Astringency, Body, Texture, Warmth-Alcohol, Sapidity/Savouriness/Saltiness, Minerality, Smoothness*

Legend: Descriptors reported in grey were not used by the judges during the tasting and therefore not shown in results tables.

Table S3. Basic physicochemical parameters of the analysed wines.

Sample		pH	Total acidity (g/L)	Citric acid (g/L)	Tartaric acid (g/L)	Malic acid (g/L)	Lactic acid (g/L)	Acetic acid (g/L)	Succinic acid (g/L)	Fructose (g/L)	Residual Sugars (g/L)	Glycerol (g/L)	Ethanol (% v/V)	Total dry extract (g/L)[#]	Net dry extract (g/L)[#]
2021	TIM01	3.06±0.00	7.4 ± 0.1	0.78±0.00	3.59±0.01	1.07±0.01	0.10±0.00	0.48±0.00	0.73±0.00	3.69±0.01	4.95±0.02	9.87±0.02	14.48±0.00	31.0	26.1
	TIM02	3.38±0.00	5.3 ± 0.1	0.38±0.00	2.85±0.01	0.18±0.01	1.48±0.01	0.34±0.00	0.92±0.00	1.29±0.01	2.01±0.00	7.82±0.03	14.00±0.01	21.4	19.4
	TIM03	3.14±0.00	5.9 ± 0.1	0.40±0.00	3.19±0.00	1.28±0.02	0.11±0.03	0.33±0.00	0.76±0.00	0.50±0.00	0.50±0.00	6.88±0.10	13.36±0.01	20.1	19.6
	TIM04	3.13±0.00	5.6 ± 0.1	0.33±0.01	2.14±0.01	1.34±0.04	0.90±0.00	0.29±0.00	0.72±0.00	0.47±0.00	0.47±0.00	7.32±0.02	13.74±0.00	19.0	18.5
	TIM05	3.08±0.00	6.0 ± 0.1	0.28±0.01	3.45±0.02	1.07±0.02	0.11±0.00	0.31±0.01	0.67±0.00	1.95±0.00	3.89±0.02	6.90±0.00	13.09±0.00	24.5	20.6
	TIM06	3.15±0.00	5.9 ± 0.0	0.39±0.01	3.31±0.03	0.81±0.01	0.24±0.00	0.30±0.00	0.78±0.00	1.53±0.01	2.13±0.83	7.76±0.00	14.78±0.04	18.3	16.2
	**2021**	**3.15±0.11**	**6.0 ± 0.7 AB**	**0.43±0.18**	**3.09±0.43 A**	**0.96±0.43**	**0.49±0.55**	**0.34±0.07**	**0.76±0.08**	**1.57±1.12**	**2.32±1.80**	**7.76±1.11**	**13.91±0.65**	**22.4±4.8**	**20.1±3.3**
2020	TIM07	3.21±0.00	5.0 ± 0.0	0.44±0.01	2.63±0.01	0.15±0.04	1.03±0.00	0.28±0.00	0.59±0.00	1.73±0.00	2.00±0.03	6.54±0.01	13.94±0.03	19.6	17.6
	TIM08	3.20±0.00	6.6 ± 0.1	0.44±0.00	3.57±0.01	1.17±0.00	0.13±0.00	0.29±0.00	0.85±0.00	0.18±0.00	0.18±0.00	10.44±0.01	14.20±0.02	25.8	25.6
	TIM09	3.31±0.00	5.5 ± 0.1	0.42±0.00	2.78±0.02	0.36±0.02	1.35±0.01	0.37±0.00	0.91±0.00	1.08±0.01	1.33±0.35	7.71±0.02	13.99±0.03	21.1	19.8
	TIM10	3.10±0.00	5.9 ± 0.0	0.37±0.00	2.81±0.01	0.77±0.06	0.54±0.00	0.25±0.00	0.74±0.00	0.24±0.00	0.24±0.00	8.67±0.02	14.03±0.01	23.2	23.0
	TIM11	3.38±0.00	5.1 ± 0.1	0.02±0.00	2.20±0.03	0.30±0.00	0.95±0.00	0.54±0.00	0.84±0.00	0.50±0.00	0.72±0.17	8.95±0.03	14.18±0.01	23.2	22.5
	TIM12	3.20±0.00	6.2 ± 0.1	0.03±0.00	3.04±0.01	1.47±0.06	0.45±0.00	0.33±0.00	0.67±0.00	0.45±0.00	0.45±0.00	9.98±0.02	13.39±0.02	26.1	25.7
	**2020**	**3.23±0.10**	**5.7 ± 0.6 AB**	**0.29±0.21**	**2.84±0.47 A**	**0.70±0.53**	**0.74±0.43**	**0.34±0.10**	**0.77±0.12**	**0.70±0.60**	**0.82±0.71**	**8.71±1.44**	**13.96±0.29**	**23.2±2.6**	**22.3±3.2**
2019	TIM13	3.09±0.00	6.4 ± 0.0	0.04±0.00	2.55±0.03	1.58±0.04	0.42±0.00	0.42±0.00	0.84±0.00	1.10±0.01	1.10±0.01	10.63±0.04	14.42±0.05	24.9	23.8
	TIM14	3.19±0.00	6.8 ± 0.0	0.43±0.01	3.69±0.01	1.32±0.01	0.24±0.16	0.28±0.00	0.79±0.00	0.30±0.00	0.30±0.00	8.77±0.10	14.83±0.01	21.6	21.3
	TIM15	3.13±0.00	6.2 ± 0.0	0.59±0.00	3.28±0.01	0.23±0.01	0.85±0.00	0.52±0.00	0.77±0.00	5.18±0.01	5.31±0.01	7.82±0.00	14.13±0.00	25.0	19.7
	TIM16	3.21±0.00	5.8 ± 0.1	0.36±0.00	3.01±0.01	1.20±0.01	0.32±0.00	0.40±0.00	0.68±0.00	0.22±0.00	0.22±0.00	7.31±0.00	14.22±0.02	20.6	20.4
	TIM17	3.12±0.00	5.9 ± 0.0	0.33±0.01	2.60±0.02	1.52±0.03	0.39±0.00	0.28±0.00	0.72±0.00	2.75±0.00	2.75±0.00	6.99±0.01	13.71±0.02	22.9	20.2
	**2019**	**3.15±0.05**	**6.2 ± 0.4 A**	**0.35±0.20**	**3.03±0.53 A**	**1.17±0.55**	**0.44±0.23**	**0.38±0.10**	**0.76±0.06**	**1.91±2.09**	**1.94±2.14**	**8.30±1.46**	**14.26±0.41**	**23.0±2.0**	**21.1±1.6**
2018	TIM18	3.24±0.00	5.6 ± 0.1	0.11±0.01	2.18±0.00	0.19±0.00	1.28±0.01	0.41±0.00	0.88±0.01	1.45±0.01	2.56±0.01	7.79±0.03	14.46±0.06	nd	nd
	TIM19	3.19±0.00	6.4 ± 0.5	0.11±0.01	3.14±0.00	1.11±0.04	0.50±0.00	0.26±0.00	0.69±0.00	0.15±0.00	0.15±0.00	8.01±0.01	14.36±0.03	24.5	24.4
	TIM20	3.11±0.00	6.1 ± 0.1	0.14±0.12	2.90±0.13	0.97±0.01	0.32±0.18	0.34±0.00	0.73±0.00	1.63±0.00	2.86±0.00	7.50±0.10	13.51±0.02	24.0	21.1
	TIM21	3.13±0.00	6.1 ± 0.1	0.15±0.01	2.79±0.00	0.71±0.18	1.28±0.00	0.41±0.00	0.94±0.00	0.22±0.00	0.22±0.00	9.65±0.01	13.26±0.02	27.9	27.7
	TIM22	3.25±0.00	6.2 ± 0.1	0.77±0.02	2.71±0.01	0.86±0.09	0.65±0.00	0.30±0.00	0.85±0.00	0.54±0.00	0.54±0.00	8.69±0.01	14.40±0.01	19.6	19.1
	TIM23	3.29±0.00	5.6 ± 0.1	0.03±0.00	2.26±0.03	2.19±0.29	0.44±0.00	0.14±0.00	0.61±0.00	2.36±0.00	2.36±0.00	6.61±0.00	13.96±0.02	23.7	21.3
	**2018**	**3.20±0.07**	**6.0 ± 0.4 AB**	**0.22±0.27**	**2.66±0.37 A**	**1.00±0.66**	**0.74±0.41**	**0.31±0.10**	**0.78±0.12**	**1.06±0.89**	**1.45±1.27**	**8.04±1.04**	**13.99±0.51**	**23.9±3.0**	**22.7±3.4**
2017	TIM24	3.15±0.00	5.4 ± 0.0	0.29±0.00	2.23±0.00	0.87±0.01	0.12±0.00	0.34±0.01	0.83±0.00	3.71±0.01	3.71±0.01	7.62±0.15	14.89±0.14	24.0	20.3
	TIM25	3.08±0.00	5.6 ± 0.1	0.27±0.00	2.47±0.00	1.11±0.01	0.13±0.00	0.24±0.00	0.79±0.00	2.50±0.00	2.50±0.00	7.00±0.01	13.92±0.00	22.7	20.2
	TIM26	3.16±0.00	5.5 ± 0.1	nd	2.65±0.01	1.25±0.01	nd	0.46±0.00	0.66±0.00	3.66±0.00	3.66±0.00	7.48±0.00	15.31±0.00	20.6	16.9
	TIM27	3.37±0.00	4.8 ± 0.1	nd	1.93±0.02	0.48±0.02	1.64±0.00	0.49±0.00	0.55±0.00	2.14±0.00	2.06±0.01	7.06±0.00	14.38±0.01	21.4	19.3
	**2017**	**3.19±0.13**	**5.3 ± 0.3 AB**	**0.14 ±0.16**	**2.32±0.29 A**	**0.93±0.34**	**0.47±0.78**	**0.38±0.11**	**0.71±0.12**	**3.00±0.80**	**2.98±0.83**	**7.29±0.30**	**14.62±0.60**	**22.2±1.5**	**19.2±1.6**
2016	TIM28	3.20±0.00	5.3 ± 0.1	0.15±0.01	2.12±0.01	0.17±0.03	1.09±0.00	0.36±0.01	1.08±0.01	0.42±0.00	0.47±0.03	9.64±0.02	15.06±0.01	18.5	18.0
	TIM29	3.26±0.00	5.1 ± 0.1	0.24±0.00	2.25±0.01	0.30±0.01	0.68±0.04	0.42±0.01	0.90±0.01	1.58±0.00	1.53±0.01	8.64±0.05	15.28±0.08	19.6	18.1
	**2016**	**3.23±0.05**	**5.2 ± 0.2 B**	**0.19±0.06**	**2.18±0.08 A**	**0.23±0.09**	**0.88±0.24**	**0.39±0.04**	**0.99±0.10**	**1.00±0.82**	**1.00±0.75**	**9.14±0.70**	**15.17±0.15**	**19.1±0.8**	**18.1±0.0**
2015	TIM30	3.21±0.00	5.0 ± 0.1	0.24±0.01	2.06±0.01	1.95±0.04	0.11±0.00	0.16±0.00	0.78±0.00	1.79±0.00	1.79±0.00	6.68±0.01	14.25±0.01	20.3	18.5
	TIM31	3.26±0.00	5.4 ± 0.0	nd	1.58±0.01	1.08±0.02	1.52±0.00	0.43±0.01	0.70±0.00	3.54±0.00	3.54±0.00	7.09±0.01	14.59±0.20	nd	nd
	**2015**	**3.23±0.03**	**5.2 ± 0.2 AB**	**nd**	**1.82±0.27 A**	**1.52±0.62**	**0.82±0.81**	**0.30±0.15**	**0.74±0.05**	**2.66±1.23**	**2.66±1.23**	**6.88±0.29**	**14.42±0.24**	**-**	**-**
	**All**	**3.19 ± 0.09**	**5.8 ± 0.6**	**0.27±0.21**	**2.71±0.54**	**0.94±0.54**	**0.62±0.49**	**0.35±0.10**	**0.77±0.11**	**1.58±1.32**	**1.82±1.48**	**8.06±1.19**	**14.20±0.56**	**22.6±3.0**	**20.9±2.9**
	**Sign. Sample**	*	***	**	***	*	ns	ns	ns	**	*	*	**		
	**Sign. Vintage**	ns	*	ns	*	ns	ns	ns	ns	ns	ns	ns	ns		

Data are expressed as average value ± standard deviation of two measurements. For ‘vintages’ these are average values of the samples ± standard deviations. Sign.: *, **, ***, and “ns” indicate significant differences at $p < 0.05$, 0.01, 0.001, and not significant, respectively, according to Kruskal-Wallis for the factor ‘Sample’ and ‘Vintage’. Different uppercase Latin letters within each column indicate significant differences among ‘Vintage’ according to Dunn test ($p<0.05$).

Table S4. Sensory results for 0-10 continuous scale evaluation of colour, typicity and liking.

Vintage	Sample	*Visual colour hue*	*Colour typicity*	*Aroma typicity*	*In-Mouth typicity*	*Liking*
2021	TIM01	7.33±1.77bcd	6.72±1.92ab	5.96±2.09abcd	5.61±2.45abc	86.4±5.08abc
	TIM02	5.55±1.69defghijk	6.22±2.37ab	5.59±2.18abcd	4.98±1.57abc	83.6±4.98abcd
	TIM03	4.54±1.61jk	6.46±1.41ab	5.45±2.04abcd	5.55±1.97abc	82.7±5.23abcd
	TIM04	3.71±1.97k	5.59±2.02b	4.66±2.47de	5.08±2.59abc	82.8±7.63abcd
	TIM05	5.10±2.05efghijk	6.83±1.89ab	6.41±1.57abcd	5.78±1.95abc	83.8±4.73abcd
	TIM06	4.92±1.47ghijk	6.94±1.55ab	5.06±2.32cd	5.21±2.41abc	84.1±5.62abc
	**2021**	**5.20±2.06C**	**6.46±1.90B**	**5.53±2.15B**	**5.37±2.14B**	**83.9±5.64AB**
2020	TIM07	5.04±1.68efghijk	7.11±1.32ab	6.71±1.87abcd	5.87±2.02abc	86.1±5.24abc
	TIM08	4.96±1.80fghijk	6.94±1.58ab	5.75±2.10abcd	5.23±1.49abc	83.1±5.08abcd
	TIM09	5.52±1.55defghijk	6.92±1.51ab	5.89±2.32abcd	5.96±1.92abc	83.9±5.71abc
	TIM10	5.95±1.60cdefghij	7.55±1.80ab	6.35±3.00abcd	5.67±2.28abc	84.4±5.59abcd
	TIM11	9.62±0.46a	2.69±2.07c	2.14±1.38e	2.26±1.22d	76.9±6.56d
	TIM12	5.82±1.65defghijk	7.64±1.29ab	7.33±2.30abcd	6.44±2.27abc	86.9±5.92abc
	**2020**	**6.15±2.19AB**	**6.48±2.33B**	**5.72±2.72B**	**5.32±2.28B**	**83.6±6.43AB**
2019	TIM13	7.05±1.50bcdef	7.94±1.41a	6.49±1.79abcd	5.52±1.85abc	82.8±4.10abcd
	TIM14	6.57±1.71bcdefghij	7.86±1.35a	6.94±2.08abcd	6.20±2.17abc	85.1±5.52abcd
	TIM15	6.91±1.40bcdefgh	7.89±1.18a	5.48±1.96abcd	4.81±1.97bc	78.8±7.28cd
	TIM16	6.04±1.60cdefghij	8.10±1.09a	7.34±1.30abc	7.25±1.89ab	87.3±4.38abc
	TIM17	6.21±1.81cdefghij	7.73±1.42a	7.66±1.44ab	7.13±1.65ab	86.0±5.62abcd
	**2019**	**6.55±1.63AB**	**7.90±1.27A**	**6.78±1.86A**	**6.18±2.09A**	**84.0±6.17AB**
2018	TIM18	6.82±1.56bcdefg	7.99±1.30a	7.91±1.49a	7.38±1.61a	87.4±5.64a
	TIM19	8.39±1.04ab	6.62±2.25ab	5.63±2.16abcd	5.99±2.02abc	81.4±4.72bcd
	TIM20	4.61±1.48ijk	7.19±1.34ab	6.34±1.35abcd	5.61±1.85abc	84.9±5.12abcd
	TIM21	7.30±1.72bcd	7.04±1.89ab	5.34±2.65bcd	5.36±2.65abc	81.0±6.28bcd
	TIM22	7.84±1.44abc	7.58±1.76ab	6.39±2.12abcd	6.54±1.78abc	85.9±5.32abcd
	TIM23	4.74±1.85jk	7.26±1.44ab	6.24±2.11abcd	5.81±2.30abc	83.6±5.51abcd
	**2018**	**6.60±2.10AB**	**7.28±1.71A**	**6.32±2.14AB**	**6.14±2.12A**	**84.1±5.81AB**
2017	TIM24	4.66±1.66jk	7.64±1.28ab	7.67±1.38ab	6.92±1.71abc	88.2±6.13ab
	TIM25	4.58±1.66hijk	7.24±1.40ab	6.95±1.88abcd	7.03±1.90ab	86.2±4.77abc
	TIM26	7.15±1.25bcde	7.29±1.69ab	5.58±1.91abcd	5.02±2.15abc	81.8±5.69bcd
	TIM27	7.08±1.53bcd	7.71±1.55a	7.31±1.78abc	6.34±2.31abc	86.4±7.19abc
	**2017**	**5.85±1.96BC**	**7.47±1.46A**	**6.90±1.88A**	**6.38±2.11A**	**85.7±6.34A**
2016	TIM28	6.62±1.48bcdefghi	7.76±1.37a	5.61±1.69abcd	5.54±1.99abc	82.1±4.43abcd
	TIM29	7.44±1.41abcd	7.40±1.74ab	5.93±2.16abcd	5.32±2.28abc	81.9±4.80abcd
	**2016**	**7.03±1.49A**	**7.58±1.56A**	**5.76±1.91AB**	**5.43±2.10AB**	**82.0±4.54B**
2015	TIM30	4.93±1.95ghijk	7.84±1.39a	7.60±1.54abc	6.72±2.10abc	87.0±4.80abc
	TIM31	7.91±1.18abc	7.32±1.81ab	4.73±2.07	4.50±2.26cd	81.6±5.80bcd
	**2015**	**6.34±2.21AB**	**7.58±1.61A**	**6.26±2.30AB**	**5.68±2.42AB**	**84.4±5.90AB**
**Sign. Sample**		***	***	***	***	***
**Sign. Vintage**		***	***	***	***	*

Data are expressed as average value ± standard deviation (n = 20 for *Visual colour hue* and *Liking* and n=18 for *Colour typicity*, *Aroma typicity*, *In-Mouth typicity*). Sign.: *, **, ***, and "ns" indicate significant differences at $p < 0.05$, 0.01, 0.001, and not significant, respectively, according to two-way ANOVA with 'Sample x Judges' or 'Vintage x Judges', with 'Judges' with random factor. Different lowercase Latin letter within each column indicates significant differences among 'Sample' according to Tukey HSD (p<0.05), whereas different uppercase Latin letters within the same column indicate differences among 'Vintage' according to Tukey HSD (p<0.05).

Table S5. Pearson Correlation among Colour, Typicity and Liking scales.

Pearson correlation	Visual colour hue	Colour typicity	Aroma typicity	In-Mouth typicity	Liking
Visual colour hue		-0.208^{ns}	-0.356*	-0.370*	-0.488**
Colour-typicity	-0.208^{ns}		0.810***	0.748***	0.565***
Aroma-typicity	-0.356*	0.810***		0.936***	0.857***
In Mouth-typicity	-0.370*	0.748***	0.936***		0.848***
Liking	-0.488**	0.565***	0.857***	0.848***	

Figure S1. Example of the proposed tasting sheet.

**Giudice 1** (Judge 1)	**Codice** (Code):

**Colore**<br>Colour hue	├──────┤<br>*verdolino* (*green*) … *paglierino* (*pale yellow*) … *dorato* (*gold*)
**Tipicità Colore**<br>Colour typicity	├──────┤<br>*0* … *10*
**Tipicità Aroma**<br>Aroma typicity	├──────┤<br>*0* … *10*
**Tipicità in bocca**<br>In-mouth typicity	├──────┤<br>*0* … *10*
**Aroma orto- e retro-nasale (descrittori)**<br>Orthonasal and retronasal aroma (descriptors)	
**Sensazione in bocca (descrittori)**<br>In-mouth sensations (descriptors)	
**Gradimento**<br>Liking	______/100

Legend: Colore/ Colour hue = *Visual colour hue in the manuscript. Tasting sheet was proposed in Italian (black) and here translated in English (grey).*

Figure S2. Pearson correlation plot of visual color characteristics and colorimetric instrumental measurements. Symbols *, **, *** indicate significant differences at $p < 0.05, 0.01, 0.001$ correlation according to t-test.

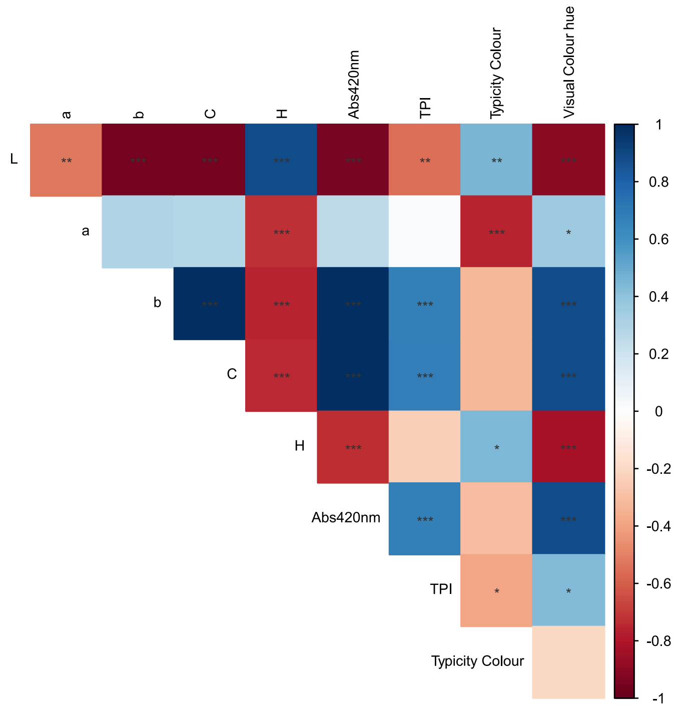

Figure S3. Correspondence Analysis (CA) of the individual descriptors (frequency >1%).

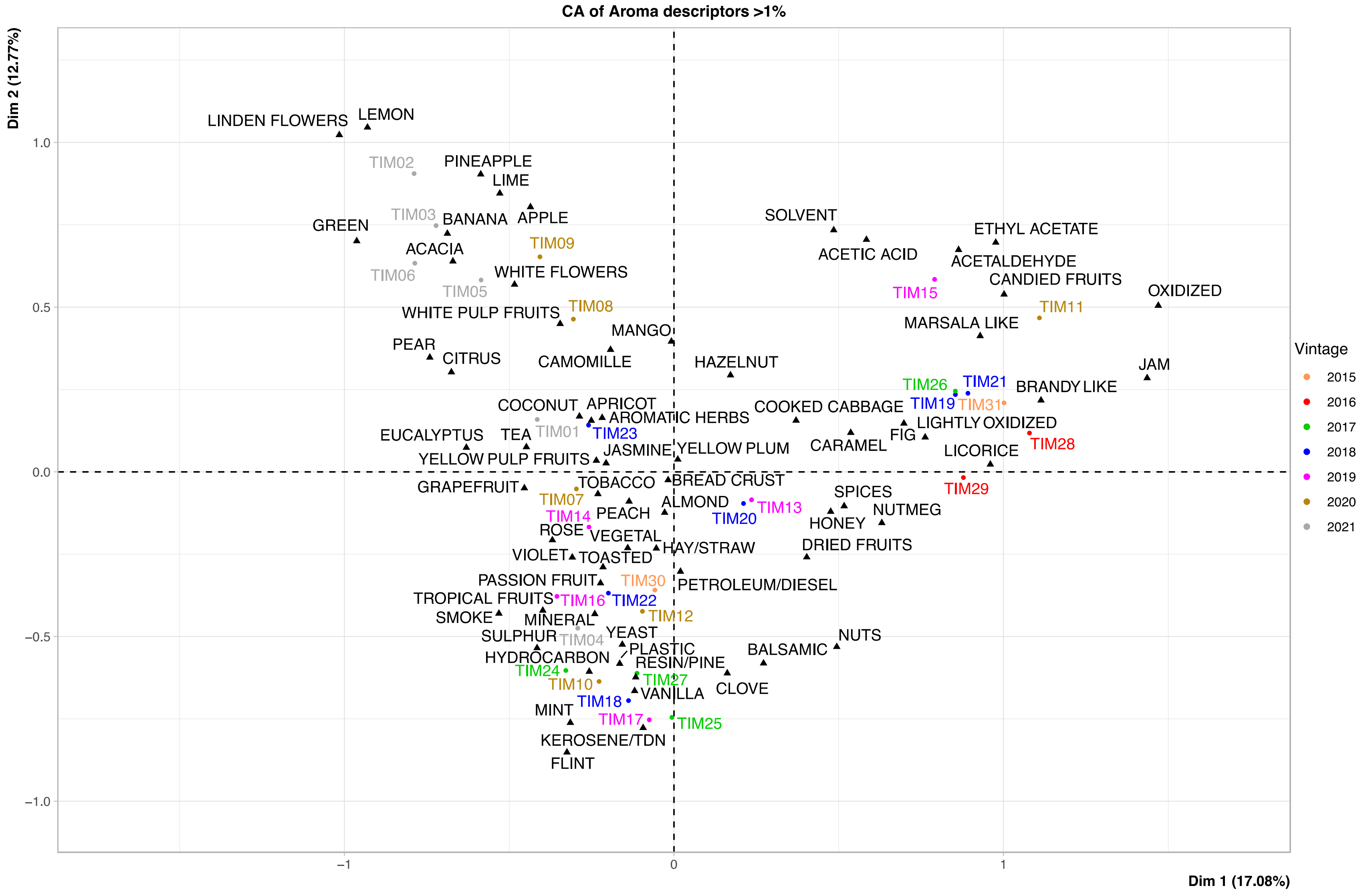

Supplement: Supplementary file 1 [file foods-14-00591-s001.zip › foods-3425125-supplementary.pdf]
